# Generation of a Fully Human scFv that binds Tumor-Specific Glycoforms

**DOI:** 10.1038/s41598-019-41567-6

**Published:** 2019-03-25

**Authors:** Zhongpeng Lu, Kalika Kamat, Blake P. Johnson, Catherin C. Yin, Nathalie Scholler, Karen L. Abbott

**Affiliations:** 1 University of Arkansas for Medical Sciences, Department of Biochemistry and Molecular Biology, Little Rock, AR 72205 USA; 2 Ouachita Baptist University, Department of Biology, Arkadelphia, AR 71998 USA; 3 SRI International Biosciences Division, Center for Cancer and Metabolism, Menlo Park, CA 94025-3493 USA

## Abstract

Tumor-specific glycosylation changes are an attractive target for the development of diagnostic and therapeutic applications. Periostin is a glycoprotein with high expression in many tumors of epithelial origin including ovarian cancer. Strategies to target the peptide portion of periostin as a diagnostic or therapeutic biomarker for cancer are limited due to increased expression of periostin in non-cancerous inflammatory conditions. Here, we have screened for antibody fragments that recognize the tumor-specific glycosylation present on glycoforms of periostin containing bisecting N-glycans in ovarian cancer using a yeast-display library of antibody fragments, while subtracting those that bind to the periostin protein with glycoforms found in non-malignant cell types. We generated a biotinylated form of a fully human scFv antibody (scFvC9) that targets the bisecting N-glycans expressed by cancer cells. Validation studies *in vitro* and *in vivo* using scFvC9 indicate this antibody can be useful for the development of diagnostic, imaging, and therapeutic applications for cancers that express the antigen.

## Introduction

Tumor cells typically display tumor-specific changes in glycosylation on surface glycoproteins and glycolipids that can serve as biomarkers for diagnosis as well as candidates for immunotherapy^1–4^. Such changes in glycosylation are due to altered expression levels of unique glycosyltransferases and glycoproteins that lead to their surface expression and potential secretion from tumor cells. However, this area of research has been hampered by having only a few specific anti-carbohydrate antibodies useful for targeting tumor cell-specific changes in glycosylation.

One approach to develop such specific anti-carbohydrate antibodies is yeast display. These technologies can improve the affinity and specificity of recognition reagents^5–7^. In this method, recombinant antibodies are displayed on the yeast surface as a fusion protein to a cell wall component (Aga-2) and library generation is facilitated by the homologous recombination system inherent in yeast^8,9^. Coupling flow cytometry with cell surface display of recombinant antibodies expressed as single chain Fragment variables (scFv) permits the monitoring of both scFv expression at the yeast surface and scFv binding to the antigen^10^. Yeast display has also proven to be highly effective for various directed evolution applications^11–15^. These methods translate into time-and cost-efficient production and screening of scFvs that have enabled the identification of many functional scFvs directed toward numerous medically relevant proteins, including scFv directed against mesothelin^16^, TEM1^17^, mannose receptor^18^, glypican^19^, and B7-H4^20^.

We have utilized the powerful advantages of the yeast display method to isolate scFv that recognize the tumor-specific bisecting glycan structures discovered in ovarian cancer^3^. These glycans are generated in part by a unique glycosyltransferase GnT-III, encoded by the *Mgat3* gene, which creates bisecting complex-type N-glycans by addition of a β1-4-linked GlcNAc to the core β-mannose of N-glycans^21^. We previously discovered that the *Mgat3* gene was highly amplified in ovarian cancer^22^. The *Mgat3* gene is amplified in several human cancers due to hypomethylation changes in the promoter near the transcription start site^23^. The structures of bisecting N-glycans in ovarian cancer are different than those bisecting N-glycans found in non-malignant cells. Unexpectedly, the bisecting N-glycans from ovarian cancers show reduced branching, lack of galactose and sialic acid, with or without core fucose making this glycan structure a biomarker for ovarian cancer and possibly several other human cancers^3^.

Our laboratory has used a targeted glycoproteomic approach to identify glycoproteins that carry tumor-associated bisecting glycan structures in ovarian cancer. Our analysis of secreted and membrane proteins from primary ovarian cancer tissues led to the discovery of periostin, also known as osteoblast-specific factor 2 (OSF-2) as a potential biomarker^3,24^. Periostin is a secreted glycoprotein that is present in circulation and also associates with the cell membranes evidenced by the presence of periostin in membrane fractions by proteomic analysis^3^. The likely mechanism of cell surface binding is due to presence of FAS1 domains that have been demonstrated to interact with the membrane in the protein fasciclin^25^. Despite the elevated levels of periostin in human cancers, this glycoprotein has not been utilized as a biomarker due to variable expression in inflammatory conditions^26–28^. This complicates the use of the protein itself as a biomarker for cancer because detection of the periostin protein levels may not correlate with the disease burden. The ability to detect the cancer-specific bisecting glycoform on periostin would be a superior biomarker for diagnostic applications and may lead to the development of new therapeutic approaches. Here, we describe our subtraction/selection process to identify a yeast-displayed scFv (scFvC9) and characterization of its specificity for tumor-specific bisecting glycan structures. We further validate the use of scFvC9 to target ovarian cancer xenograft tumors *in vivo*. Together these finding suggest the potential use for this antibody in diagnostic and therapeutic applications for cancers that have amplification of the *Mgat3* gene.

## Materials and Methods

### Cell lines

Periostin cDNA cloned into a retroviral vector was a gift from Dr. Xiao-Fan Wang (Duke University, Durham, NC). Virus was produced using 293-GP2 packaging cells and the VSV-G envelope prior to transduction into recipient cells (Lec4, Pro5, OVCAR3) to create periostin (PN) expressing cell lines used for depletions and enrichments. The CHO cell lines Lec4 and Pro5 were gifts from Dr. Pamela Stanley (Albert Einstein College of Medicine, Bronx,NY). The OVCAR3 and OVCA26 control and GnT-III shRNA cell lines have previously been described^3,29^. Human mesothelin A1847, C30, and human mesothelin Luc-ID8 cell lines were generated by Dr. Scholler (SRI International, Menlo Park, CA).

### Western blot analysis

Cell culture supernatant (50 mL) was collected from OVCAR3-PN, Pro5-PN, and Lec4-PN cells with the addition of protease inhibitors. Periostin was purified on anti-Flag resin (Sigma-Aldrich) according to the manufacturer instructions. Proteins were separated on NuPage 4–12% BisTris gel using 1X MES buffer prior to transfer to PVDF membrane. Blots were blocked in 3% BSA/1X TBST before detection of bisecting glycans using (1:5,000) dilution of biotin labeled E-PHA (Vector Labs) and (1:10,000) dilution of streptavidin HRP (Vector Labs) followed by enhanced chemiluminescent detection. The blot was stripped in Pierce (Thermo) stripping buffer, blocked in 5% nonfat milk 1XTBST and detected using (1:250) dilution of antibody to periostin (Santa Cruz Biotechnologies).

### Selection of bisecting glycan-selective scFv by screening a yeast-display scFv library

A yeast display library of scFvs isolated from infiltrating B cells and PBMCs derived from 11 ovarian cancer patients has been previously described^30^. This library was grown in SD-CAA (0.67% yeast nitrogen base, and 0.5% Casamino acids) and the induction of cell surface display of scFv was induced as previously described^31^. Multiple rounds of library depletion were performed as follows: 1 × 10^8^ induced yeast-display scFv in phosphate buffered saline (PBS) were added to PBS rinsed adherent Lec4-PN cells (95% confluent T175 flask). Non-adherent yeast after 30 min of incubation were taken to another T175 flask of Lec4-PN cells and this process was repeated for a total of 6 flasks. This process was repeated using Pro5-PN flasks. Next, this new depleted sub-library was grown and induced again and used to enrich for scFv binding to the tumor-specific glycosylation on periostin using the OVCAR3-PN cells. Following 6 rounds of enrichment with manual selection of bound yeast using a cell selector probe the level of enrichment was monitored using yeast-cell ELISA as follows: Yeast in the scFv enriched pool were spread on SD-CAA plates and allowed to grow for 2–3 days to allow colonies to develop. Individual colonies were streaked onto separate SD-CAA plates and induced with SGR-CAA to allow scFv expression on the yeast cell surface. Yeast scFv were labeled using fluorescent brightener 28 (Sigma-Aldrich, calcofluor) 1 mg/mL in H_2_O/NaOH. Briefly, yeast with scFv on the cell surface were resuspended at 1 × 10^7^ in calcofluor solution (10% final) for 5 min at room temperature followed by washes in PBS. Labeled yeast were panned on Lec4-PN/Pro5-PN/OVCAR3-PN cells at 90% confluence on 24-well plates for 30 min at room temperature. Differential yeast binding to cells were measured with an Envision 2104 multilabel reader at (Ex355/Em405) before and after each 5 minutes wash with gentle shaking. Post wash readings were made following removal of wash buffer and addition of fresh PBS.

### Transformation of yeast-display scFv into soluble scFv

ScFv DNA was PCR amplified from lysed yeast. Briefly, 5 μL of yeast grown at saturation were suspended in 20 μL of 20 mM NaOH and microwaved 3 min, to lyse yeast. DNA corresponding to the scFv fragment was amplified by PCR using Phire DNA polymerase and gel purified prior to cotransformation with linearlized p416BCCP vector into the VYH10 yeast strain by electroporation^17^. Yeast were grown overnight in SD CAA media supplemented with tryptophan (TRP) and further induced in 1 mL of SGR CAA/TRP as previously described^6^. Soluble scFv were confirmed using an ELISA assay using the HIS and V5 tags for detection. Soluble scFv clones were transformed into site-specific biotinylated soluble antibodies (biobodies) as described previously^32^.

### ADCC Assay

OVCAR5 cells (3 wells per condition) were plated at 0.8 × 10^4^ cells/well 48 hrs prior to addition of scFvC9 antibody alone, anti-myc antibody alone, or serial dilutions of scFvC9 mixed with anti-myc antibody. Complexes with scFvC9 at 0.5 mg/mL and anti-myc antibody at 1 mg/mL were formed at 4 °C for 30 min. prior to addition to cells. Serial dilutions of complexes and control scFvC9 alone (0.5 mg/mL or anti-myc antibody alone (1 mg/mL) were added to cells for 48 hrs. Equal volume of CellTiter-Glo Reagent (Promega) was added to each well. The plate was shaken on an orbital shaker for 2 minutes and placed at room temperature for 10 minutes prior to recording luminescence. The resulting cell lysis generates luminescent signal proportional to ATP present in the number of viable cells. Three independent experiments were performed.

### Immunochemistry Cell Staining

Ovarian cancer cells were plated on poly L-lysine coverslips and grown to 50% confluence prior to immunofluorescent staining. scFvC9 biobody antibody (50 μg/mL) in PBS was added to cells for 5 min. or 30 min. time points. Cells were washed with PBS before fixation in ice cold methanol for 5 min. Cells were blocked with PBS/1% BSA for 10 min. before detection of scFvC9 biobody using streptavidin conjugated Alexa Fluor 594. Nuclei were counterstained with a 1:10,000 solution of DAPI for 10 seconds before mounting in Vectashield media.

### Xenograft scFvC9 Imaging

Immune compromised NSG female mice were injected subcutaneously with 1.0 × 10^6^ A1847 human ovarian cancer cells six wk before imaging studies. Immune competent C57Bl/6 female mice were injected intraovary or intraperitoneal with 1.0 × 10^6^ luciferase transduced ID8 murine ovarian cancer cells 8 wk prior to imaging^33^. Luc-ID8 tumors were monitored with luciferin injections prior to the imaging study. Mice were anesthetized with isoflurane and imaged prior to antibody injection for baseline and then at the 2 min., 5 min., 30 min., 60 min., 4 hr, 24 hr, and 48 hr time points after injection of antibody complexes. The scFvC9 complexes included 30 μg scFvC9 biobody pre-incubated with 1:1 fluorescently labeled streptavidin IRB680W for 30 min at 4 °C to form complexes. IV injection of complexes was performed retro orbitally for all mice.

### Immunofluorescence Localization of scFvC9 in Tissues

NSG mice bearing subcutaneous A1847 tumors were injected with 30 μg scFvC9 biobody and sacrificed 24 hr later to harvest tumor, kidney, spleen, lung, and liver. All tissues were immediately fixed in formalin and stored in 70% ethanol until tissue section. Slides were deparaffinized by sequentially dipping in xylene and grated ethanol series. Tissue was incubated with Streptavidin-Qdot 800 (diluted 1:50) in PBS for 1 hr at room temperature in the dark. Slides were washed 3 times in PBS/0.05% tween 20 and counterstain was performed with DAPI at 1:10,000 for 15 min. Slides were washed 2X in PBS and fluorsave reagent was used to mount the slides.

### Magnetic Resonance Imaging

#### *In Vitro* Analysis

MRI imaging was performed on a 1.5 T MR system (Bruker PharmaScan 70/16). Phantom tubes were generated with A1847, C30, or ID8 cells (0.4–1 × 10^6^ cells) layered between spacers of agarose gel before or after incubation with 25 μg/mL or 50 μg/mL of scFvC9 coupled to anti-flag magnetic beads. The scFvC9/magnetic bead complexes were incubated with the cells for 30 min at 4 °C before washing and fixing with 2% paraformaldehyde for 20 minutes at 4 °C. Fixed cells and scFvC9/magnetic complexes were then resuspended in 100 μL in 1% agarose gel and finally layered between spacers of 2% ultralow gel temperature agarose to generate phantom tubes. Optimal T1 and T2 weighted sequences were determined and regions of interest for each cell layer were measured for control cells only and control magnetic beads only for comparison with cells incubated with C9/magnetic beads. Results from three separate experiments were calculated and the ± SEM for normalized signal intensities were calculated.

#### *In Vivo* Analysis

MRI imaging was performed on a 1.5 T MR system. NSG mice bearing 6 wk subcutaneous A1847 xenograft tumors were injected with avidin-coated magnetic beads only or scFvC9 biobody coupled 1:2 with avidin-coated magnetic beads in 100 uL of PBS for 30 minutes at 4 °C. Regions of interest were calculated for tumor and control (muscle) across each 2 mm slice. The temperature during MR imaging was 28 °C and the time of acquisition was 30 min. Signal intensity (SI) values of tumor were divided by control (muscle) to yield the normalized signal intensity. Normalized signal intensities were calculated before and 1 hr, 4 hr, or 24 hr following magnetic bead only or C9/magnetic bead injections via retro orbital injection.

### Animal Study Ethics

All animal studies and procedures were conducted under a protocol approved by the SRI International Institutional Animal Care and Use Committee. All methods were performed in accordance to guidelines and regulations at SRI International. SRI International maintains a centralized animal care and use program registered with the U.S. Department of Agriculture (USDA), accredited by the Associatiion for Assessment and Accreditation of Laboratory Animal Care International (AAALAC) and has an assurance on file with the Office of Laboratory Animal Welfare (OLAW).

## Results

### Selection of human scFvs binding with tumor-specific glycans

**Figure 1:**
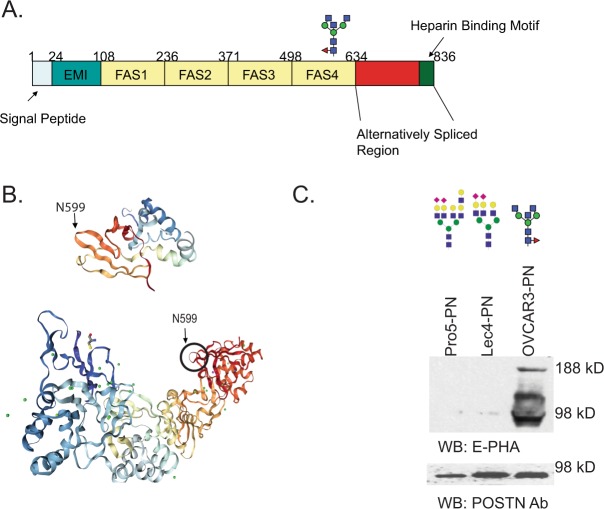
Periostin domain structure and location of complex N-linked glycosylation. (**A**) Domain map of the human periostin protein with the glycosylation site in the last FAS1 domain marked. (**B**) NMR structure (PDB 5WT7) of the FAS4 domain showing the unstructured loop where asparagine 599 is located^35^. Crystal structure (PDB 5YJG) of the FAS1-FAS4 domains for human periostin with the N599 solvent exposed^34^. (**C**) Western blot analysis of periostin protein purified from culture supernatant on anti-Flag resin. The top cropped image is detected using the lectin E-PHA (Vector Labs) and the bottom cropped image is the detection of the same blot with periostin antibody (Santa Cruz Biotechmologies). Examples of previously detected glycan structures for each cell line are shown above each lane^3,39^.

Periostin has one highly conserved N-linked glycosylation site located in the last FAS1 domain near the C-terminus of the protein (Fig. 1A). The functions of the glycosylation present on periostin are not known; however, this site is highly conserved in sequence implying its potential importance, and this site is present in all known isoforms of periostin. The conservation and location of the N-glycosylation site in an unstructured, solvent exposed region^34,35^ (Fig. 1B) led us to the hypothesis that we could use the perostin protein as a scaffold to display different glycoforms of periostin allowing subtraction and enrichment of specific scFv antibodies to glycoforms of periostin. The top image displays the NMR structure of the last FAS1 domain of human periostin (Fig. 1B) indicates that asparagine 599 (the amino acid that is glycosylated) is located in the unstructured loop. The location of this region within the crystal structure of all FAS1 domains is shown in the bottom image further validating the exposure of the N-glycosylation site. There are three main forms of N-glycans: high mannose-type, hybrid-type, or complex-type. Typical glycoproteins have several N-glycosylation sites that can consist of any of these three forms. It is not yet well understood why certain sites have a tendency to be high mannose and other sites are hybrid or complex. However, prior research studies indicate there is site specificity for these glycan forms within glycoproteins^36^. We have determined that the single N-glycosylation site in periostin displays complex N-glycans due to the glycosylation pattern changes in different cancers. Our previous glycoproteomic analysis of breast cancer tissues indicates that periostin displays tetra-antennary sialylated complex N-linked glycans^37^. In ovarian cancer tissues our previous studies indicate that periostin displays truncated, agalactosylated, asialylated N-glycan structures with or without core fucose^3,24^. Despite the high expression in human cancer tissues, human cancer cell lines grown under adherent growth conditions do not express periostin. Cell lines that are grown under non-adherent conditions permit the formation of spheroids that begin to express periostin. We created stable periostin expression in the ovarian cancer cell line OVCAR3 as well as the non-malignant Chinese hamster ovary (CHO) cell lines Pro5 (parental) and Lec4 (lacking GnT-V expression) to allow expression under adherent growth conditions. As shown in Fig. 1C, periostin isolated using a Flag tag antibody bound to the lectin E-PHA, a lectin known to recognize bisecting N-glycans^38^, only for the OVCAR3-PN cell line indicating the presence of bisecting glycans. There are additional higher molecular weight bands reacting bound by E-PHA indicating that other glycoproteins were isolated with periostin that also carry this form of glycosylation in ovarian cancer cells. The Pro5-PN and Lec4-PN flag tag pull downs are negative for E-PHA binding reflecting an absence of bisecting glycosylation in these cell lines (Fig. 1C). Previously published mass spectrometry analysis of N-glycosylated glycoforms found on glycoproteins isolated from Pro5 and Lec4 cells lines suggests that tetra-antennary and tri-antennary complex-type N-glycans are prominent in these cell lines^39^. All cell lines express similar levels of periostin protein (Fig. 1C). These results confirm that periostin is expressed in these cell lines with different forms of complex-type N-glycans enabling us to use these for the subtraction and selection of scFv antibodies.

**Figure 2:**
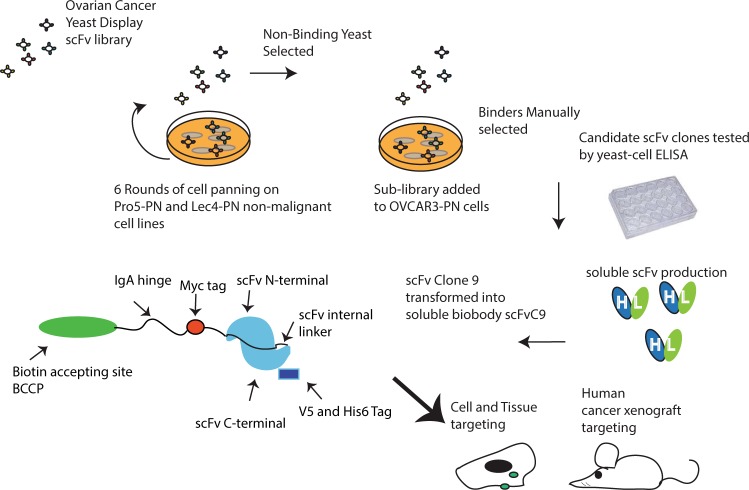
Schematic flow of the selection, purification, and validation approach. The ovarian cancer yeast-display scFv library was first subtracted using 6 rounds each on the non-malignant Pro5-PN and Lec4-PN cells. Non-binders were grown and added to OVCAR3-PN cells for multiple rounds of selection. Clonal populations of binders were evaluated using yeast-cell ELISA and yeast that had binding specificity for bisecting glycans were made into secreted scFv. Clone 9 was converted to a biotin labeled antibody known as a biobody with the indicated tags and evaluated using cell lines and xenograft tumor models.

**Figure 3:**
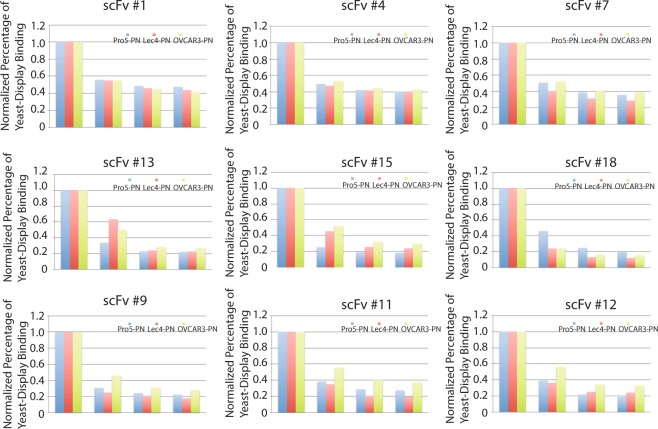
Representative yeast-cell ELISA results. Differential binding of candidate clonal yeast populations were measured on Pro5-PN (blue), Lec4-PN (red), and OVCAR3-PN (green) cells plated at 90% confluence on 24-well plates. Bound yeast (labeled with Calcofluor) were measured before and after washes. Representative data shown reflect the percentage of yeast bound after each wash for each cell line for the indicated clones.

The scFv yeast-display library used was isolated from the B cells of ovarian cancer patients. Our enrichment strategy described in Fig. 2 consists of multiple rounds of subtraction using the Pro5-PN and Lec4-PN cell lines to create a new sub-library that is then added to OVCAR3-PN cells to select binding yeast clones. Yeast-display binding clonal populations (n = 21) were further screened by panning onto adherent OVCAR3-PN, Lec4-PN, and Pro5-PN cells using a yeast cell-ELISA procedure. Figure 3 shows a representative analysis of scFv binding clones using these cell lines. Certain clones such as #1, #4, and #7 bound with similar affinity to all cell lines following sequential washes suggesting that these clones do not demonstrate specificity and affinity for any cell line; other clones such as #13, #15, and #18 bound best to Lec4-PN and OVCAR3-PN (#13 and #15) or Pro5-PN (#18) indicating these scFv clones show affinity to protein elements or glycan elements that are not ovarian cancer specific. However, other clones such as #9, #11, and #12 differentially bound with affinity to OVCAR3-PN cells following each sequential wash indicating specificity for ovarian cancer. Clone #9, #11, and #12 were transformed into soluble scFv antibody as previously described^17^.

### *In vitro* analysis of scFvC9 binding specificity, distribution, and antibody-initiated cytotoxicity

**Figure 4:**
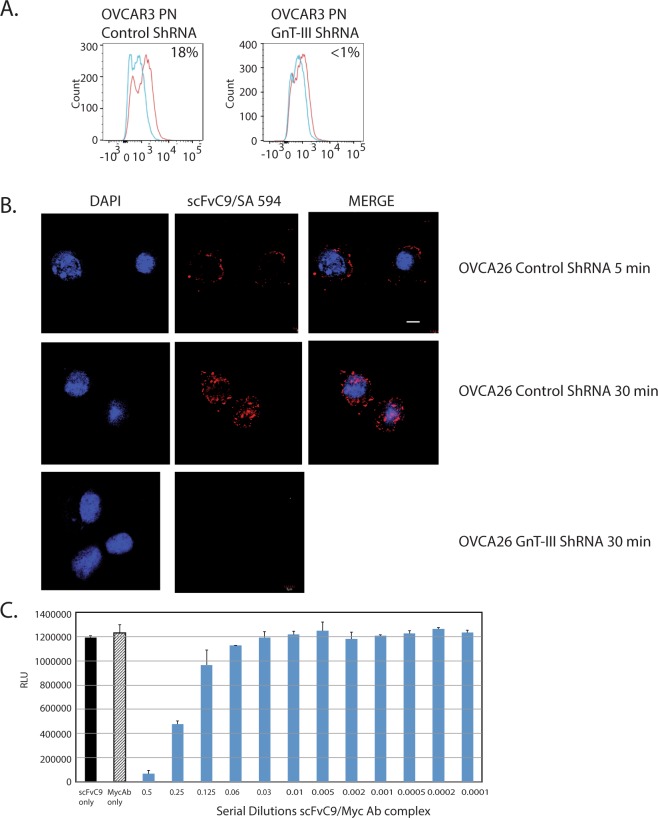
Specificity, cellular localization and antibody-dependent cytotoxicity for scFvC9 biobody. (**A**) Flow cytometry analysis of OVCAR3-PN Control ShRNA and OVCAR3-PN GnT-III ShRNA cells stained with scFvC9 biobody premixed with streptavidin APC (red lines) or streptavidin APC onlty (blue lines). (**B**) Representative images of scFvC9 biobody binding and internalization into OVCA26 cells, bar 10 μm. (**C**) Functional analysis of cell cytotoxicity using a cell titer glow luminescence viability assay. ScFvC9 biobody was premixed with anti-myc mAb and serial dilutions were added to cell for 48 hr at 37 °C. The results shown are representative of 3 independent experiments.

Clone #9 had the optimal yields of soluble biotin labeled scFv antibody production and was further analyzed for binding specificity to bisecting N-glycans using OVCAR3 cells. We established stable periostin expression in OVCAR3 cells that have stable expression of control ShRNA not targeting any gene or ShRNA targeting GnT-III (*Mgat3* gene)^3,29^. Flow cytometry data shown in Fig. 4A show that scFvC9 has increased binding to control OVCAR3-PN cells compared with GnT-III ShRNA OVCAR3-PN cells indicating binding specificity for bisecting N-glycans. OVCAR3-PN Control ShRNA and OVACR3-PN GnT-III ShRNA both express periostin protein indicating that the binding is specific to the bisecting N-glycan and not the protein. Next, to evaluate the potential targeting and internalization of scFvC9 we used microscopy to track the binding and distribution of scFvC9 in ovarian cancer cells using the patient-derived cell line OVCA26 previously described^29^. Cell staining of OVCA26 Control ShRNA cells at 5 min indicates an accumulation of scFvC9 at the cell surface (Fig. 4B). The antibody is fully internalized at the 30 min time point. We observed no binding of scFvC9 to OVCA26 GnT-III ShRNA cells further validating the specificity for bisecting N-glycans. We have further evaluated the binding of scFvC9 to glioblastoma cells since this tumor type also has elevated levels of GnT-III expression. Our 30 minute binding data shown in Supplementary Fig. 1 shows that scFvC9 binds to control LN18 cells that display bisecting glycans and there is no binding to LN18 Crispr/Cas9 KO of Mgat3 (SFig. 1B). These data confirm that scFvC9 requires the bisecting glycan for binding and that bisecting structures from other cancers can be targeted. The accumulation of scFvC9 at the cell surface suggests that scFvC9 may be capable of functional in initiating antibody-dependent cell cytotoxicity. The scFvC9 biobody contains a myc tag (Fig. 2) allowing us to expose cells to scFvC9/anti-myc ab complexes to evaluate cytotoxicity. The ovarian cancer cell line OVCAR5 was premixed with serial dilutions of antibody complexes for 48 hrs before cell viability was measured using a luminescent viability assay. The results indicate that scFvC9 alone or anti-myc ab alone did not induce cytotoxicity (Fig. 4C). However, exposure of the cells to the first two serial dilutions of the complex (2.5 μg/mL and 1.25 μg/mL) had cytotoxic activity.

### Targeting, stability, and specificity of scFvC9 for tumors *in vivo*

**Figure 5:**
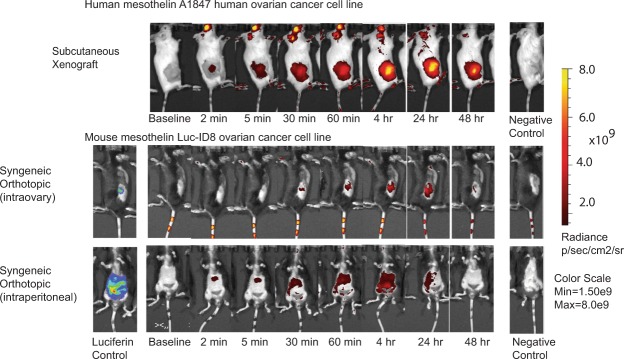
IVIS imaging of ovarian tumors. Top panel: immune compromised NSG female mice with 6 wk subcutaneous xenograft tumor from A1847 human ovarian cancer cells were imaged before and after retro-orbital injection of scFvC9/IRB680W complexes or negative control (IRB680W only). Middle panel: immune competent C57Bl/6 female mice with 8 wk intraovary Luc-ID8 murine ovarian cancer cells were imaged before and after injection of scFvC9/IRB680W complexes or negative control 9IRB680W only). Lower panel: immune competent C57Bl/6 female mice with 8 wk intraperitoneal Luc-ID8 murine ovarian cancer cells were imaged before and after scFvC9/IRB680W complex injection or IRB680W only.

We used *in vivo* imaging (IVIS) to evaluate the ability of scFvC9 to target tumors *in vivo* using both human xenograft and syngeneic mouse models. The top panel of Fig. 5 shows the localization and accumulation of scFvC9 complexed 1:1 with fluorescent-labeled streptavidin in NOD/Scid mice with human A1847 ovarian cancer subcutaneous xenograft tumors established 6 wk prior. The scFvC9 antibody targets the tumor and accumulates in the tumor with a peak at 24 hrs and a gradual decline beginning at 48 hrs. Next, we evaluated the ability of scFvC9 to target luciferase transduced ID8 murine ovarian cancer cells (Luc-ID8) in the immune competent C57Bl/6 female mice. Cells were injected intraovary or intraperitoneal 8 wk prior to IVIS imaging. The scFvC9 antibody complexed 1:1 with fluorescent-labeled streptavidin was injected retro orbitally at the indicated times prior to imaging. The intraovary injections (Fig. 5 middle panel) accumulated at the maximum in the 24 hr time point as observed for the human subcutaneous xenograft injections (Fig. 5, top panel). However, the decline at 48 hr was more substantial. The syngeneic intraperitoneal model reached a maximum accumulation of scFvC9/fluorescent streptavidin complexes at the 4 hr time point. These results confirm that scFvC9 can target both human and mouse ovarian tumors *in vivo* by retro orbital injection.

**Figure 6:**
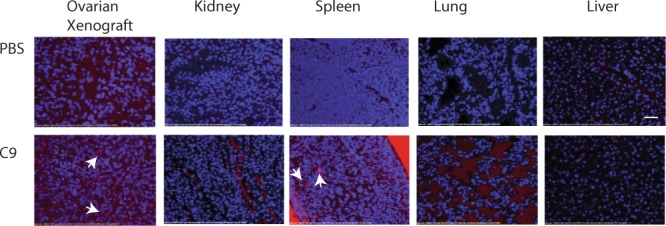
Detection of scFvC9 biobody in tumors and tissues at the 24 hr time point. Immune compromised female NSG mice with subcutaneous A1847 xenograft tumors were injected with scFvC9 biobody IV 24 hr before necropsy and tissue collection. Sections were stained with Streptavidin Qdot (1:50 in 1X PBS) prior to counterstain with DAPI. White arrows mark regions of interest discussed in the text, Bar 100 μm.

To evaluate the specificity of the scFvC9 antibody for tumor tissues and not normal tissues we evaluated antibody localization following injection. NSG mice bearing A1847 subcutaneous tumors were injected with scFvC9 biobody or vehicle only. Mice were sacrificed 24 hr later and tissues were harvested for immunofluorescent staining with streptavidin Qdot 800 to localize the scFvC9 biobody. We observed very punctate signals localized to the periphery of the nuclei in the tumor cells indicative of endosomal compartment localization (Fig. 6 first image lower panel white arrow marks examples). The kidney, an organ known to express non-malignant bisecting N-glycans was negative for the punctate epithelial cell staining of scFvC9 seen in the tumor. While we do observe staining in the blood vessel of the kidney, the epithelial cells of the kidney tissue were negative. Some background staining could be seen in the spleen; but this staining can be observed in areas between nuclei suggesting possible extracellular localization (Fig. 6 third image lower panel arrows show examples) rather than accumulation of the antibody perinuclear as observed with tumor cells (Fig. 4B) and tumor tissue (Fig. 6 first image lower panel). We also notice some accumulation of scFvC9 in the extracellular spaces in the lung. Overall, the scFvC9 antibody demonstrates the ability to preferentially target malignant epithelial cells *in vivo* via the vasculature.

### Magnetic resonance imaging (MRI) validation studies

**Figure 7:**
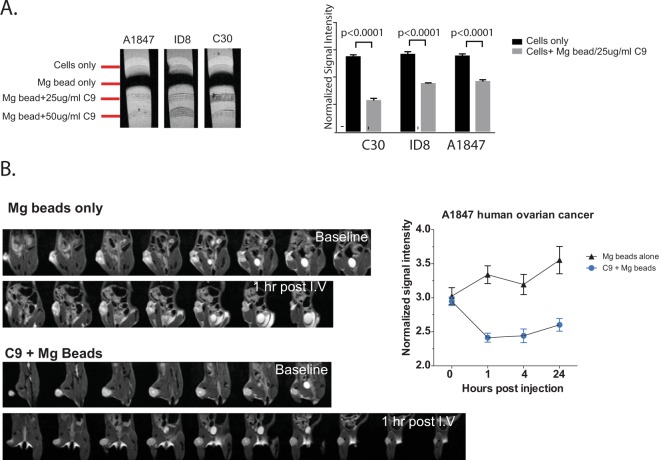
MR studies with scFvC9 biobody. (**A**) Phantom tubes layered with cells only, anti-flag magnetic bead only, or cells with scFvC9 biobody and anti-flag magnetic beads were MR imaged. Representative image shown and results in graph to the right represent mean decreased signal intensity from 3 independent experiments, ±SEM P < 0.0001. (**B**) Immune compromised NSG female mice with A1847 subcutaneous tumor were injected with scFvC9 coupled 1:2 with magnetic avidin beads. Representative 1 hour images are shown and cumulative normalized (SI tumor/SI muscle for given ROI) signal intensity for each time point are graphed to the right.

Due to the successful targeting of tumors with scFvC9 we tested whether the scFvC9 biobody could target magnetic beads to the tumor for amplification of signal by magnetic resonance imaging studies. Successful development of scFvC9 as a targeted MR imaging probe would require specificity, magnitude of accumulation, and stability. We started the evaluation of scFvC9 as an MR imaging probe by measuring the ability to detect scFvC9 magnetic bead complexes in ovarian cancer cells *in vitro* by MRI using phantom tubes. A1847, ID8, and C30 cells were embedded in agarose and layered. Layers of cells only (washed and fixed), anti-flag tag magnetic beads only, or cells (incubated with scFvC9/anti-flag tag magnetic beads prior to wash and fix) were measured using MRI. The results demonstrated a significant reduction of normalized signal intensity was detectable in the layers containing cells with scFvC9/magnetic bead versus cells alone (Fig. 7A). These results illustrate the accumulation of signal amplification.

Next, we initiated subcutaneous A1847 xenograft tumors in NSG mice to test the ability of scFvC9/magnetic bead complexes to target tumors *in vivo*. Avidin-coated magnetic beads alone or complexed 1:2 with site-specific biotinylated scFv (C9 biobody) were injected IV retro-orbitally. Animals were MR imaged before and at 1 hr, 4 hr, or 24 hr post injection. Regions of interest (ROI) for tumor and control (muscle) were measured across a 2 mm slice. The normalized signal intensity differences between magnetic beads alone and scFvC9/magnetic bead complexes were highly significant at all post injection time points (Fig. 7B). Representative images from the 1 hr post injection time point are shown (Fig. 7B). These data illustrate that scFvC9/magnetic bead complexes have specificity to target tumor and show signal amplification, specificity, and stability as the reduction in MRI signal for the tumor was consistent from the 1 hr to 24 hr time points.

## Discussion

Our results indicate the successful development of an effective screening platform that led to the isolation and purification of a fully human scFv antibody scFvC9 that targets a prominent tumor-specific glycosylation change. We characterized the binding specificity and targeting of this antibody for ovarian cancer and our initial microscopy data using the LN18 glioblastoma cell line indicate that scFvC9 should bind other tumors that exhibit amplification of the *Mgat3* gene^23^. We have developed the scFvC9 clone into a biobody allowing large scale purification and demonstrated the specificity of scFvC9 biobody for tumor glycans *in vitro* and *in vivo*. The cell surface binding and internalization of the antibody with enhanced stability *in vivo* are qualities that should enable future development of diverse imaging and therapeutic applications. The scFvC9 biobody could be conjugated to diverse therapeutic molecules such as immune-conjugates, toxins, or drug-conjugates. In addition to these potential therapeutic innovations; the biobody can be useful for tumor imaging and potentially pairing of imaging and therapy options.

Most antibodies developed against tumor antigens target protein despite the fact that there are numerous well-known tumor carbohydrate antigens such as the Tn, sialyl-Tn, Thomsen-Friedenreich (TF), LeX, sialyl-LeX, and LeY^40^. Antibodies that have been isolated to many of these tumor-glycan epitopes are IgM leading to limited applications in clinical use. The isolation of antibodies against membrane protein glycoforms or secreted protein glycoforms from human patient-derived antibody libraries has been limited and this may be due to lower abundance of antibodies that target these antigens within the libraries. Therefore, we employed new strategies in this study to overcome this limitation allowing the isolation of a fully human scFv that targets a prominent tumor-glycan (scFvC9). The repeated subtractions of a patient-derived library with an intact glycoprotein expressing non-tumor glycoforms prior to antigen enrichment using the intact glycoprotein expressing tumor-glycoforms is a key component of our strategy. Our use of mammalian cells to screen the library rather than purified glycoprotein or synthetic synthesized glycopeptides is also unique. To our knowledge, this is the first isolation and description of a human scFv that targets a complex-type N-linked tumor glycan.

Single-chain antibodies have been utilized previously to select for antibodies against glycans. Most of the previously published studies utilized phage-display rather than yeast-display. Yeast antibody libraries display posttranslational modifications similar to mammalian cells and this may offer advantages in solubility and folding. Phage-display was used to isolate human single-chain antibodies toward the glycolipid carbohydrate antigen G(M3) with specificity for melanoma and breast cancer cells *in vitro*^41^. Another study using phage-display demonstrated that human single-chain antibodies that target sialyl-LeX and LeX could be isolated from a patient-derived library^42^. The most famous tumor carbohydrate antigens, Tn and STn, present a challenge due to the smaller size of these carbohydrate antigens. Single-chain antibodies that target the Tn antigen were isolated due to a strategy that included construction of a mouse scFv library from mice immunized with Jurkat cells that display prominent Tn and STn antigens along with a coordinated subtraction and enrichment strategy led to the isolation of scFv targeting the Tn antigen^43^. Our strategy builds on these studies utilizing the following features: (i) we have screened a patient-derived scFv library developed from the B cells of 11 different ovarian cancer patients (from peripheral blood lymphocytes and ascites) increasing the depth of the library, (ii) we have panned our library using mammalian cells expressing a glycoprotein that displays the tumor-glycans allowing optimal presentation of the tumor glycan, (iii) we utilized multiple rounds of subtraction and enrichment, and (iv) our method uses complementary yeast systems that permit the production of cell surface scFv and secreted scFv with similar conformations minimizing changes in antibody binding specificity.

We are confident that scFvC9 binds tumor-specific bisecting N-glycoforms and is not dependent on periostin protein expression due to our yeast cell-ELISA data, flow cytometry analysis, and cell staining; however, we do not know at this time the exact structures of the N-glycoforms that scFvC9 is binding. The antibody was isolated using a human ovarian cancer cell line that may express differences in the bisecting N-glycoforms from the structures we have previously determined from primary ovarian cancer tissues^3^. Our validation analysis using human ovarian cancer cell lines (OVCA26, C30, A1847), murine ovarian cancer cells (ID8-Luc), and human glioblastoma cells (LN18) that are each distinct from the OVCAR3 cell line that was used to isolate the antibody add confidence that scFvC9 recognizes a broad range of tumor bisecting N-glycans.

There are powerful advantages for antibodies that recognize tumor-glycans. Patients make antibodies against tumor-associated antigens, including glycans. It is known that tumor-specific glycoforms on proteins can overcome immune tolerance^44^. Attempts to elicit humoral immune response to MUC1 peptides failed; yet chemoenzymatically synthesized MUC1 peptide with cancer associated O-glycan Tn and STn epitopes elicited a cancer-specific humoral response^44^. Antibodies that target tumor-glycans may work with checkpoint inhibitors to improve strategies to overcome the immune suppression for solid tumors. Antibodies that target tumor-glycans could improve targeted chemotherapy strategies due to the abundance of the tumor carbohydrate antigen on multiple proteins. Single-chain antibodies to tumor glycans, due to the small size, can be developed into novel therapeutics for glycoproteins that may not have been thought of as traditional drug targets. In summary, our results demonstrate a new approach useful for the isolation of human antibodies that target tumor-specific glycans.

## Supplementary information

LaTeX Supplementary File

## Data Availability

No datasets were generated or analysed during the current study.
