# Supplementary material for: Generation of a Fully Human scFv that binds Tumor-Specific Glycoforms
Source: Sci Rep. 2019 Mar 25;9:5101. doi: 10.1038/s41598-019-41567-6 (PMC6433917; doi:10.1038/s41598-019-41567-6)
Supplement: Supplementary file 1 — LaTeX Supplementary File [file 41598_2019_41567_MOESM1_ESM.pdf]

Supplement Fig. 1

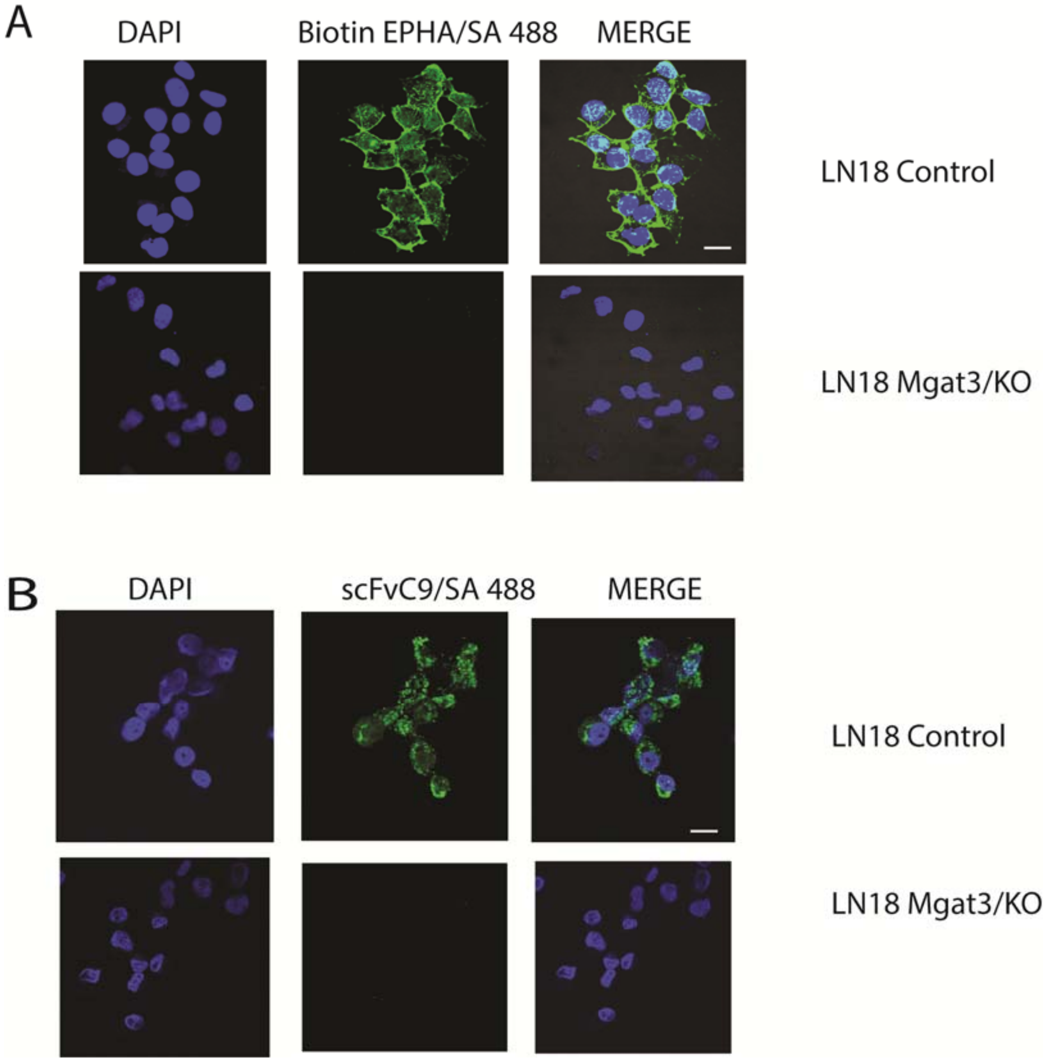

Supplement Fig. 1 scFvC9 cell binding and specificity in human glioblastoma cells. (A) Crispr/Cas9 KO of the Mgat3 gene in single cell isolated LN18 clone known as C2 is confirmed by the absence of E-PHA binding indicating a loss of bisecting N-glycan. The non-targeted single cell isolated clone known as control A1 has Mgat3 expression confirmed by the binding of E-PHA lectin., bar 20 μm (B) The scFvC9 biobody binds to LN18 Control A1 clone and has no binding to the LN18 Crispr/Cas9 Mgat3 KO clone C2..
